# Yet another acetate in the wall — RWA-C regulates wood xylan acetylation in poplar

**DOI:** 10.1093/plphys/kiad442

**Published:** 2023-08-05

**Authors:** Dyoni M Oliveira

**Affiliations:** Assistant Features Editor, Plant Physiology, American Society of Plant Biologists; Department of Plant Biotechnology and Bioinformatics, Ghent University, Ghent, 9052, Belgium; VIB Center for Plant Systems Biology, Ghent, 9052, Belgium

Wood biomass provides the largest form of terrestrial carbon storage and is a renewable and potentially carbon-neutral feedstock for pulping, biofuels, and a variety of bio-products. Increasing biomass use is a strategy to mitigate the energy crisis and climate change challenges. Bottlenecks due to incomplete use of biomass components are still common in biorefineries, however. Engineering plant cell wall components for the easier processing of wood helps to overcome this challenge and maximize biomass processing (Mota et al. 2019; De Meester et al. 2022).

Cellulose, hemicelluloses (including xylans), and the phenolic polymer lignin are the main components of wood cell walls. Depolymerization of cellulose and hemicelluloses releases sugars that can be converted into high-value compounds such as biochemicals, biofuels, bioplastics, etc., although the presence of lignin limits the efficient conversion of these polysaccharides into products (Oliveira et al. 2020). In the secondary cell walls, xylan hemicelluloses cross-link cellulose and lignin, forming the lignin–carbohydrate complex. The role of modifications of the xylan structure is not completely understood; however, recent findings have shed light on functions of xylan modifications in the cell wall assembly and how such modifications are associated with improved processing of biomass (Oliveira 2023). Reductions in the acetate levels in the xylan backbone (referred to as xylan acetylation) generally make it easier to enzymatically hydrolyze the cell wall polysaccharides into fermentable sugars (Pawar et al. 2017).

In this issue of *Plant Physiology*, Zhang et al. (2023) functionally characterize a gene, *REDUCED WALL ACETYLATION-C* (*PtRWA-C*), involved in xylan acetylation in poplar (*Populus trichocarpa*) wood. The starting point for the authors’ work was a poplar population of 463 lines with transfer DNA insertions (Busov et al. 2011), which was developed to identify regulators of the secondary cell wall biosynthesis. From this population, a gain-of-function mutant was screened with a higher lignin amount and ratio of the 2 main lignin monomers — syringyl-to-guaiacyl (S/G) ratio — and *PtRWA-C* was further identified as the gene causing this lignin phenotype (Fig. 1).

By comparing publicly available gene expression data of different organs and cell types of poplar stem, the authors demonstrated that *PtRWA-C* is highly expressed in stem-differentiating xylem that at later developmental stages makes up most of the wood biomass (Tung et al. 2023). Previous findings had shown that poplar contains 4 *RWA* genes (*PtRWA-A, -B, -C*, and *-D*), which are grouped into 2 clades (RWA-A/B and -C/D) involved in wood acetylation (Pawar et al. 2017). Zhang and colleagues observed that unlike the other 3 *PtRWAs* members*, PtRWA-C* is also highly expressed in phloem and cambium regions, suggesting that PtRWA-C might have a broader function acetylating not only xylan but also other cell wall matrix components, as previously observed for its homolog, *AtRWA2*, in Arabidopsis (*Arabidopsis thaliana*) (Manabe et al. 2011).

Because the expression of genes involved in secondary cell wall biosynthesis is temporally and spatially coordinated, coexpression network analysis is a powerful strategy to pinpoint genes potentially involved in the same biological process. Therefore, Zhang and colleagues used a coexpression network analysis with expressing genes in stem-differentiating xylem to confirm that *PtRWA-C* has similar spatial-temporal expression patterns with several genes involved in secondary cell wall deposition.

What is the function of PtRWA-C in wood acetylation? The authors explored this question by overexpressing *PtRWA-C* in poplar and characterized the wood cell walls. Consistent with the previous finding of higher lignin amount and S/G ratio in the gain-of-function transfer DNA insertion mutant, *PtRWA-C* overexpressing lines had increased lignin amount and S/G ratio. Remarkably, chemical analyses of wood cell walls demonstrated a large increase (1.7-fold) in xylan acetylation of *PtRWA-C* transgenic lines. Therefore, PtRWA-C protein may function in the machinery to acetylate the xylan backbone. A second question arises of how the higher level of acetate groups in the xylan may affect the conversion of poplar wood into fermentable sugars. To answer this question, Zhang and colleagues measured the enzymatic conversion of cell wall polysaccharide of poplar stem into fermentable sugars. Both *PtRWA-C* transgenic lines showed reduced release of glucose and xylose from wood biomass compared with wild type (Fig. 1). These results suggest that boosting xylan acetylation may also trigger an increase in lignin amounts, negatively affecting the release of sugars from the *PtRWA-C* overexpressing wood.

Based on the expression-based quantitative trait loci (eQTL) analysis, the authors identified that *RWA-C* expression is regulated by *cis-*regulatory eQTL along with other transcription factors known to be involved in secondary wall biosynthesis. Next, to specifically determine the regulatory relationship between *PtRWA-C* and genes involved in secondary cell wall biosynthesis, they combined yeast 1-hybrid and dual-luciferase reporter assays to demonstrate that *PtRWA-C* is regulated by regulators of secondary cell wall biosynthesis and the stress-responsive HARDY (HRD) family transcription factor. Specifically, HRD binds to the *PtRWA-C* promoter and thereby activates *PtRWA-C* expression. These results suggest that secondary cell wall–related transcription factors and HRD directly regulate the expression of *PtRWA-C*, which could be potential targets for genetic engineering to modulate the gene expression and generate trees with improved wood properties.

In summary, Zhang et al. provide not only insights into the biological role of PtRWA-C in xylan acetylation and how it affects further biomass processing but also identify regulatory genes and cis-eQTL that regulate the expression of *PtRWA-C*. These findings may serve as potential targets for gene and promoter editing to alter the levels of gene expression and wood acetylation, ultimately changing the biomass processing towards biofuels and bioproducts.

**Figure 1.**
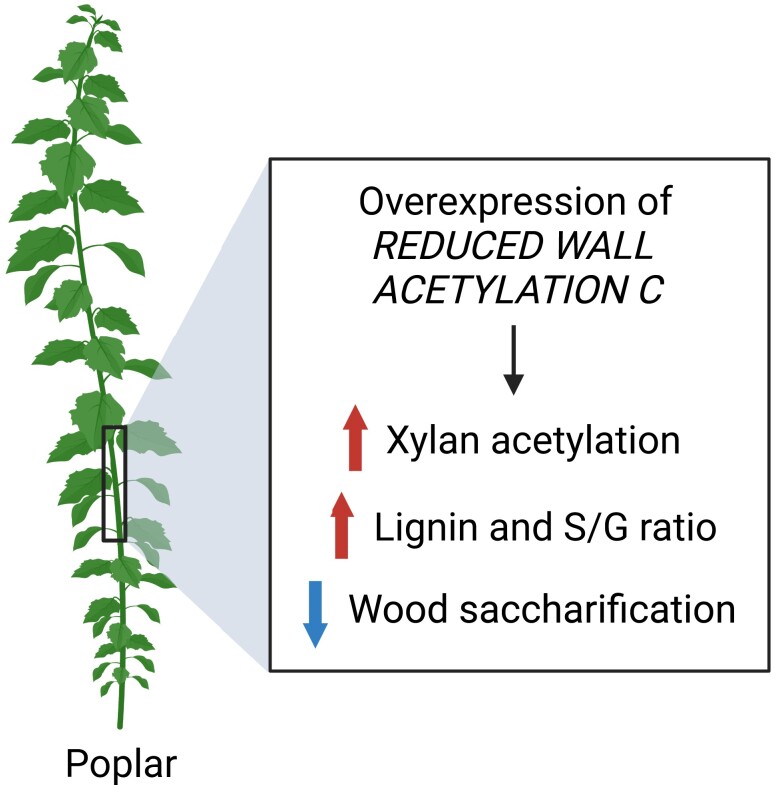
Summary of the changes in poplar cell walls by overexpressing *PtRWA-C*. Guaiacyl (G) and syringyl (S) monomeric units in lignin. Figure was created with BioRender.com.

## Data Availability

None.

## Dive Curated Terms

The following phenotypic, genotypic, and functional terms are of significance to the work described in this paper:

wood AmiGo: PO:0005848
